# Clinical implications of a novel prognostic factor AIFM3 in breast cancer patients

**DOI:** 10.1186/s12885-019-5659-4

**Published:** 2019-05-14

**Authors:** Ang Zheng, Lin Zhang, Xinyue Song, Yuying Wang, Minjie Wei, Feng Jin

**Affiliations:** 1 Department of Breast Surgery, the First Affiliated Hospital of China Medical University, 110001 No.155 Nanjing Road, Heping Districrt, Shenyang, Liaoning Province People’s Republic of China; 2 Department of Surgery, Huamei Hospital, University of Chinese Academy of Sciences (Ningbo No.2 Hospital), 315000 No.41 Xibei Road, Haishu District, NingBo, Zhejiang Province People’s Republic of China; 3 Department of Pharmacology, School of Pharmacy, China Medical University, 110122 No.77 Puhe Road, Shenbei New District, Shenyang, Liaoning Province People’s Republic of China; 4 Liaoning Key Laboratory of molecular targeted anti-tumor drug development and evaluation, China Medical University, No.77 Puhe Road, Shenbei New District, Shenyang, 110122 Liaoning Province People’s Republic of China; 5 Department of Breast Surgery, Liaoning Cancer Hospital and Institute, Cancer Hospital of China Medical University, 110042 No.44 Xiaoheyan Road, Dadong District, Shenyang, Liaoning Province People’s Republic of China

**Keywords:** AIFM3, Breast cancer, Prognosis, Homology modeling, Clinical implication

## Abstract

**Background:**

In a time of increasing concerns over personalized and precision treatment in breast cancer (BC), filtering prognostic factors attracts more attention. Apoptosis-Inducing Factor Mitochondrion-associated 3 (AIFM3) is widely expressed in various tissues and aberrantly expressed in several cancers. However, clinical implication of AIFM3 has not been reported in BC. The aim of the study is to investigate the crystal structure, clinical and prognostic implications of AIFM3 in BC.

**Methods:**

AIFM3 expression in 151 BC samples were assessed by immunohistochemistry (IHC). The Cancer Genome Atlas (TCGA) and Kaplan-Meier survival analysis were used to demonstrate expression and survival of AIFM3 signature. Gene Set Enrichment Analysis (GSEA) was performed to investigate the mechanisms related to AIFM3 expression in BC.

**Results:**

AIFM3 was significantly more expressed in breast cancer tissues than in normal tissues. AIFM3 expression had a significant association with tumor size, lymph node metastasis, TNM stage and molecular typing. Higher AIFM3 expression was related to a shorter overall survival (OS) and disease-free survival (DFS). Lymph node metastasis and TNM stage were independent factors of AIFM3 expression. The study presented the crystal structure of AIFM3 successfully and predicted several binding sites when AIFM3 bonded to PTPN12 by Molecular Operating Environment software (MOE).

**Conclusions:**

AIFM3 might be a potential biomarker for predicting prognosis in BC, adding to growing evidence that AIFM3 might interact with PTPN12.

**Electronic supplementary material:**

The online version of this article (10.1186/s12885-019-5659-4) contains supplementary material, which is available to authorized users.

## Background

On a global scale, breast cancer (BC) is the most frequent malignancy and the leading cause of cancer death among females [1]. In China, BC accounts for 12.2% of new cases diagnosed with cancer and 9.6% of cancer deaths [2]. Although ‘Escalation’ on the basis of proven treatments has resulted in better outcomes for appropriate patients, there is a still challenge that ‘De-escalation’ requires more valuable evidence and rigorous judgment [3]. Multigenic assays and some other possible ways are being used to categorize BC patients and guide systemic therapy. As ‘de-escalation’ requires more valuable evidence and rigorous judgment, filtering new prognostic factor is considered to be an effective way [4].

Apoptosis-Inducing Factor Mitochondrion-associated 3 (AIFM3) contains 598 amino acids, with two major domains. The characteristic Rieske domain localizes to the mitochondria and induced apoptosis, while pyridine nucleotide-disulfide oxidoreductase domain in the cytosol is speculated to have addition functions which have not been fully clarified [5]. Although AIFM3 is widely expressed in various tissues, the function of AIFM3 in occurrence and development progress of cancer is rarely reported. AIFM3 is aberrantly expressed only in cholangiocarcinoma (CCA) tissues, suggesting that AIFM3 can be a potential target molecule for CCA chemotherapy [6]. AIFM3 is a direct target of miR-210 which is related to proliferation and enhanced radio-sensitivity in hypoxic human hepatoma cells [7]. To date, AIFM3 has not been reported in BC, so it remains unclear whether the expression of AIFM3 is associated with the related clinical outcomes in BC patients.

Protein Tyrosine Phosphatase Nonreceptor-type12 (PTPN12) is reported to be a tumor suppressor and protective prognostic factor for BC [8]. Fundamental function for PTPN12 has been recognized in apoptotic pathway, keeping stability balance and normal function [9]. PTPN12 acts on an unidentified substrate—upstream of caspase-3 activation—to facilitate cellular detachment during apoptosis [10]. AIFM3 mediates the release of cytochrome c from the mitochondria to the cytosol and cleavage of caspase-3 [11]. Protein mass spectrum in previous work revealed that a total of 104 proteins including AIFM3 was differentially expressed between PTPN12-overexpressing HCC-1937 cell line and control group. The interaction between PTPN12 and AIFM3 in caspase-dependent apoptosis needs more evidence.

In the present study, we used bioinformatics analysis including the Cancer Genome Atlas-breast cancer (TCGA-BRCA), Kaplan-Meier survival analysis and Gene Set Enrichment Analysis (GSEA) to demonstrate expression level, survival and the mechanisms related to AIFM3 signature in BC. Then we investigated AIFM3 expression by immunohistochemistry (IHC) and explored how AIFM3 affected clinical pathology factors and patient survival in a random sample of 151 BC patients. The crystal structure of AIFM3 was modelled and intramolecular interaction of AIFM3 and PTPN12 was predicted by Molecular Operating Environment software (MOE).

## Methods

### TCGA and Kaplan-Meier survival analysis

Gene expression (https://cancergenome.nih.gov/) from TCGA-BRCA database was downloaded, containing 113 samples of normal breast tissues and 1109 samples of breast cancer tissues. Then edgR package was used to normalize gene expression in R environment. The different expression of AIFM3 in normal tissues and cancer tissues was analyzed by Graphpad Prism 7.0. Kaplan-Meier survival analysis for the relationship between survival time and AIFM3 signature was performed by Kaplan-Meier Plotter (http://kmplot.com/analysis/), an online database of published microarray datasets that assess the effect of 54, 675 genes on survival using 5, 143 breast cancer samples [12].

### Gene set enrichment analysis (GSEA)

GSEA (http://www.broadinstitute.org/gsea/index.jsp) was performed to investigate the mechanisms related to AIFM3 expression in BC patients [13]. The 1109 breast cancer samples in TCGA-BRCA were divided into high and low expression group by the median expression of AIFM3. One thousand permutations for gene sampling were used to consider statistically significant and ensure the credibility of the results. The inclusion criteria were normalized *P* < 0.05 and false discovery rate (FDR) < 25%. The annotated gene sets of version 6.0 (H, C2 and C6) were downloaded from the Molecular Signatures Database (MsigDB). GSEA was conducted based on two groups and then significant enriched pathways related to malignant tumor biological process were chosen according to normalized enrichment score (NES). Relational biological processes, cellular components and molecular functions were verified.

### Modelling crystal structure of AIFM3

MOE contains user interface enhancements for protein modeling, protein-protein interaction prediction and new scientific applications for computer-aided molecular design. Firstly, the sequence of human AIFM3 from NCBI database (accession code: Q96NN9) was downloaded and sequence similarity was searched by NCBI BLAST tool. Then, target protein sequence was aligned based on the sequence of the template and MOE 2018 package (Chemical Computing Group, Montreal, QC, Canada) was used for homology modeling. The parameters at one sidechain samples were set at the temperature of 300 K, ten mainchain models and medium intermediates refinement. The final model was scored by the Generalized Born/volume integral (GB/VI) [14]. Amber 10: EHT force field was selected for the whole modeling process. At last, energy of homology model was minimized with MOE. Ramachandran plots were used to evaluate the homology modeling of AIFM3.

### Docking AIFM3 onto PTPN12

MOE Protein-Protein Dock was used to identify the intramolecular interactions of PTPN12 and AIFM3. At first, the structure of PTPN12 was prepared by MOE QuikPrep. Then the structure of homology model of AIFM3 was opened and docked with PTPN12. According to the tutorial of MOE Protein-Protein Dock, Bead interaction energy model equals Evdw plus Eele and Egb/vi. One hundred poses of these two proteins was generated and the lowest energy pose which had the strongest binding was chosen.

### Patients and tissue samples

One hundred fifty-one patients pathologically diagnosed with infiltrative ductal carcinoma in the First Affiliated Hospital of China Medical University was evaluated. The median age of the selected patients at diagnosis was 51.3, ranging from 25 to 81. The inclusion criteria were as follows: (i) curative operations; (ii) available formalin-fixed and paraffin-embedded specimens; (iii) reliable medical records. The collected BC tissues were cut into 4 μm sections.

### Collection of clinical information

Data regarding age and tumor size were collected from Hospital Information System. The status of ER, PR, HER2, histological grade and lymph node metastases were collected from patient chart. The status of Ki67 could not be collected from patient directly, as Ki67 was not examined routinely before 2011. Herein, Pathology Department of the First Affiliated Hospital of China Medical University was invited to do an extra detection of Ki67 in all specimens of this study. Two professional pathologists who were blinded to the experiment separately evaluated IHC results. OS (Overall survival) and DFS (Disease-free survival) were collected from patients or immediate family members through telephone follow-up twice a year. OS was defined from the date of diagnosis to cancer-related death, and DFS was recorded from the date of diagnosis to the occurrence of local recurrence or distant metastasis Clinical stage relied on the clinical staging criteria set by the American Joint Committee on Cancer (AJCC).

### IHC

Streptavidin-peroxidase (S-P) method was used for staining. Firstly, the sections were de-waxed by xylene and rehydrated in graded alcohol series. Next, we retrieved the antigen under high pressure using 10 mM sodium citrate buffer (pH =6.0). Ultra-sensitive™ S-P Kit (Maixin-Bio, China) was used to block endogenous peroxidase activity and reduce non-specific reactivity. Then, the sections were incubated with primary antibody against AIFM3 (1:100 dilution, Santa, US) at 4 °C overnight, followed by incubation with secondary antibody and streptomycin avidin-peroxidase, according to protocol in Ultra-sensitive™ S-P kit. Finally, the sections were visualized with DAB reagent.

### Evaluation of IHC

Two professional pathologists who were blinded to the experiment separately evaluated DAB staining. Each slide was examined at least five times and 100 cells were observed during each examination at 400X magnification. AIFM3 expression were estimated by double score semi-quantitative analysis. Staining intensity was recorded as 0 (negative), 1 (weak), 2 (moderate) and 3 (strong). As for the percentage of positive cells, scores were marked as 0 (< 5%), 1 (6–25%), 2 (26–50%), 3 (51–75%), and 4 (> 76%). The final IHC staining score was determined by multiplying the staining intensity levels with the positive percentage staining scores. In this way, BC patients were categorized into two groups: AIFM3-high (score > 3) and AIFM3-low patients (score ≤ 3).

### Statistical analysis

In this study, all statistical analyses were performed using SPSS 24.0 (Chicago, IL, USA). The relationship between AIFM3 expression and clinical pathology factors was examined by Pearson chi-square tests, Fisher’s exact tests and logistic regression analyses. Spearman rank correlation analysis was used to show the correlation. Survival probabilities were judged by the Kaplan-Meier method and assessed by a log-rank test. OS curves and DFS curves were generated to evaluate the survival differences between the AIFM3-high and AIFM3-low patients. Cox proportional hazards regression models were used to examine the effects of AIFM3 expression on patient survival. The diagnostic value were analyzed using the ROC analysis. The area under the curve (AUC) more than 0.5 was considered to have diagnostic value. Probability values less than 0.05 were considered statistically significant.

## Results

### Expression of AIFM3 in breast cancer

**Fig. 1:**
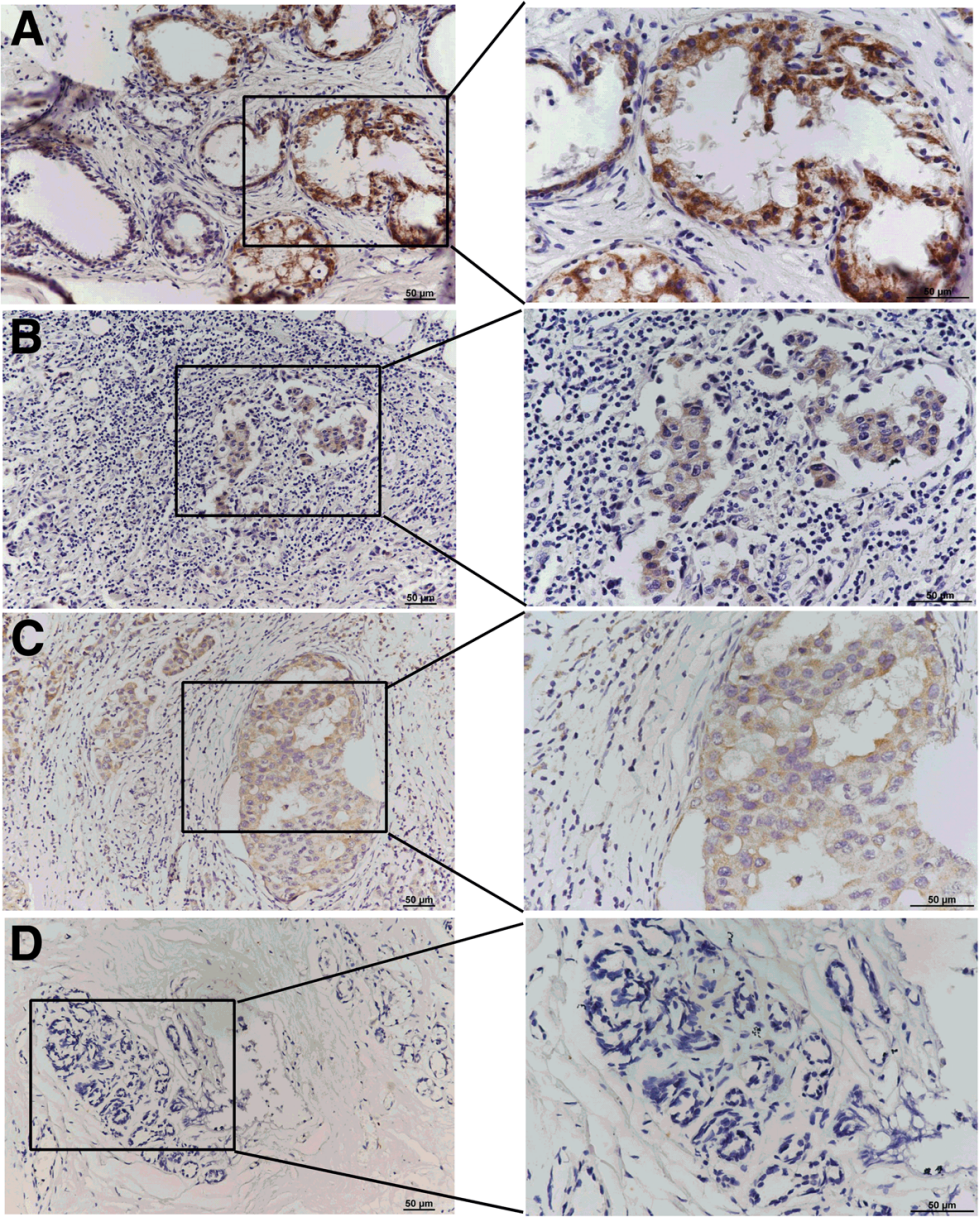
Staining range in IHC of AIFM3 (**a**) deep staining (**b**) medium staining (**c**) light staining and (**d**) no staining

To elucidate whether AIFM3 contributed to breast cancer, we evaluated the expression levels of AIFM3 by IHC in 151 real samples. We observed a wide range of staining, including no staining, light staining, medium staining and deep staining, as shown in Fig. 1a-d. IHC revealed an AIFM3 overexpressed rate of 62.9% (95/151) in BC, which was significantly higher than 30.0% (12/40) in adjacent normal breast tissues (*P* < 0.001).

### Association between AIFM3 expression and clinical pathology factors

To further elucidate how AIFM3 was involved in the breast cancer development, we analyzed the correlation of AIFM3 expression with clinical pathology factors. Univariate analysis (Table 1) illustrated the significant correlation between AIFM3 expression and tumor size (*P* = 0.013), lymph node metastasis (*P* = 0.001), molecular typing (*P* = 0.031) and TNM stage (*P* < 0.001). Multivariate analysis (Table 2) showed that lymph node metastasis (*P* = 0.015) and TNM stage (*P* = 0.009/0.003) were independent factors of AIFM3 expression.

**Table 1:** Univariate analysis of AIFM3 expression and clinical pathology factors

Factors	Number (%)	AIFM3 expression		χ^2^	*P*	Crude OR (95 CI)
		High (%)	Low (%)			
Age(years)				1.254	0.740^a^	
≤ 40	26(17.2)	14(53.8)	12(46.2)		0.318^b^	0.583(0.203-1.680)
41-50	52(34.4)	34(65.4)	18(34.6)		0.903^b^	0.944(0.376-2.375)
51-60	40(26.5)	25(62.5)	15(37.5)		0.712^b^	0.833(0.317-2.190)
≥61	33(21.9)	22(66.7)	11(33.3)			Reference
Tumor size				6.135	0.013^a^	
≥ 3 cm	87(57.6)	62(71.3)	25(28.7)		0.014^b^	2.330(1.186-4.577)
< 3 cm	64(42.4)	33(51.6)	31(48.4)			Reference
LN Metastases				11.824	0.001^a^	
negative	86(57.0)	44(51.2)	42(48.8)			Reference
positive	65(43.0)	51(78.5)	14(21.5)		0.001^b^	3.477(1.681-7.194)
ER				0.266	0.606^a^	
negative	58(38.4)	35(60.3)	23(39.7)			Reference
positive	93(61.6)	60(64.5)	33(35.5)		0.606^b^	1.195(0.608-2.349)
PR				1.160	0.281^a^	
negative	59(39.1)	34(57.6)	25(42.4)			Reference
positive	92(60.9)	61(66.3)	31(33.7)		0.282^b^	1.447(0.738-2.837)
Ki67				1.051	0.305^a^	
negative	62(41.1)	42(67.7)	20(32.3)			Reference
positive	89(58.9)	53(59.6)	36(40.4)		0.306^b^	0.701(0.355-1.384)
Her2				2.645	0.104^a^	
negative	101(66.9)	59(58.4)	42(42.6)			Reference
positive	50(33.1)	36(72.0)	14(28.0)		0.106^b^	1.831(0.879-3.811)
Histological grade				1.902	0.398^a^	
2	124(82.1)	81(65.3)	43(34.7)			Reference
3	14(9.3)	7(50.0)	7(50.0)		0.264^b^	0.531(0.175-1.612)
unrated	13(8.6)	7(53.8)	6(46.2)		0.415^b^	0.619(0.196-1.959)
Molecular typing				7.935	0.047^a^	
Luminal A	60(39.7)	41(68.3)	19(31.7)		0.011^b^	3.596(1.337-9.673)
Luminal B	48(31.8)	32(66.7)	16(33.3)		0.021^b^	3.333(1.200-9.256)
Her-2	19(12.6)	13(68.4)	6(31.6)		0.048^b^	3.611(1.012-12.888)
TNBC	24(15.9)	9(37.5)	15(62.5)			Reference
TNM staging				17.197	<0.001^a^	
I	38(25.2)	14(36.8)	24(63.2)			Reference
II	75(49.6)	50(50.0)	25(50.0)		0.003^b^	3.429(1.517-7.749)
III	38(25.2)	31(81.6)	7(18.4)		<0.001^b^	7.592(2.651-21.743)

*P*-value ^a^ came from Pearson chi-square tests or Fisher’s Exact Test

*P*-value ^b^ came from logistic regression analyses

**Table 2:** Multivariate analysis of AIFM3 expression and clinical pathology factors

Factors	Number (%)	AIFM3 expression		*P*	Adjusted OR (95 CI)
		High (%)	Low (%)		
Tumor size					
≥ 3 cm	87(57.6)	62(71.3)	25(28.7)	0.223	0.494(0.159–1.537)
< 3 cm	64(42.4)	33(51.6)	31(48.4)		Reference
LN Metastases					
negative	86(57.0)	44(51.2)	42(48.8)		Reference
positive	65(43.0)	51(78.5)	14(21.5)	0.015	4.016(1.304–12.370)
Molecular typing					
Luminal A	60(39.7)	41(68.3)	19(31.7)	0.119	2.740(0.772–9.729)
Luminal B	48(31.8)	32(66.7)	16(33.3)	0.550	1.536(0.376–6.274)
Her-2	19(12.6)	13(68.4)	6(31.6)	0.064	3.720(0.925–14.958)
TNBC	24(15.9)	9(37.5)	15(62.5)		Reference
TNM staging					
I	38(25.2)	14(36.8)	24(63.2)		Reference
II	75(49.6)	50(50.0)	25(50.0)	0.009	3.585(1.380–9.312)
III	38(25.2)	31(81.6)	7(18.4)	0.003	6.073(1.871–19.710)

**Fig. 2:**
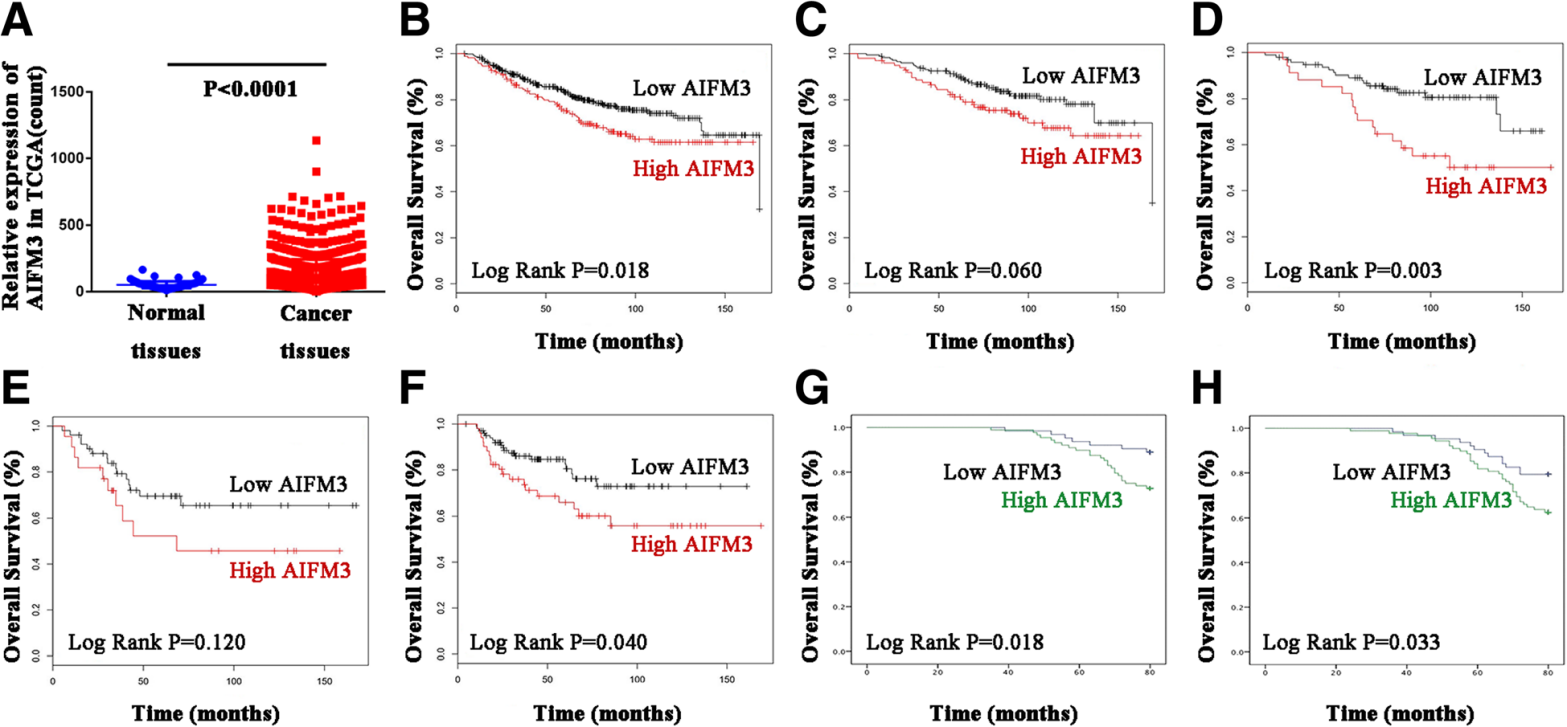
Expression and survival of AIFM3 signature in breast cancer (**a**) AIFM3 was significantly more expressed in breast cancer tissues than in normal tissues in TCGA (*P* < 0.0001). **b** High AIFM3 expression was relevant to a shorter OS in all BC patients in Kaplan-Meier plotter (*P* = 0.018). **c** High AIFM3 expression was relevant to a shorter OS in luminal A patients in Kaplan-Meier plotter (*P* = 0.060). **d** High AIFM3 expression was relevant to a shorter OS in luminal B patients in Kaplan-Meier plotter (*P* = 0.003). **e** High AIFM3 expression was relevant to a shorter OS in Her-2 type patients in Kaplan-Meier plotter (*P* = 0.120). **f** High AIFM3 expression was relevant to a shorter OS in basal-like type patients in Kaplan-Meier plotter (*P* = 0.040). **g** Overexpression of AIFM3 was significantly associated with a shorter OS in 151 patients (*P* = 0.018). **h** Overexpression of AIFM3 was significantly associated with a shorter DFS in 151 patients (*P* = 0.033)

We analyzed AIFM3 expression in TCGA datasets. The TCGA RNA Seq data demonstrated that AIFM3 was significantly over-expressed in breast cancer compared with non-cancerous tissue samples. (*P* < 0.01, Fig. 2a). A dot plot of AIFM3 levels was shown to classify “high” and “low” AIFM3-expression groups, (*P* < 0.01, Additional file 1: Figure S1).

### Correlation of AIFM3 with prognosis in breast cancer patients

Bioinformatics analysis of data mining with the Kaplan-Meier plotter was performed. Log-rank test of OS curves revealed that overexpression of AIFM3 was significantly associated with a shorter OS in BC patients (*P* = 0.018, Fig. 2b). As to various molecular typing groups, our results showed that high expression of AIFM3 was relevant to a shorter OS in luminal A patients (*P* = 0.060), luminal B patients (*P* = 0.003), Her-2 patients (*P* = 0.120) and basal-like type patients (*P* = 0.040) (Fig. 2c-f).

In 151 cases of patients, Kaplan-Meier survival analysis were used to assess the association of AIFM3 expression with OS and DFS. Using log-rank tests (Fig. 1g-h), we determined overexpression of AIFM3 was significantly associated with a shorter OS and DFS, respectively (*n* = 151. OS, *P* = 0.018, DFS, *P* = 0.033). Then we used univariate Cox regression analysis to assess the impact of each clinical pathology variable on OS and DFS in BC patients. The OS and DFS were significantly associated with the following elements: tumor size (OS, P = 0.003; DFS, *P* < 0.001), lymph node metastases (OS, *P* < 0.001; DFS, *P* < 0.001), Her2 (OS, *P* = 0.006), TNM staging (OS, *P* = 0.027/0.003; DFS, *P* = 0.022/0.002) and AIFM3 expression (OS, *P* = 0.022; DFS, *P* = 0.034) (Additional file 2: Table S1 and Table S2). Furthermore, multivariate Cox regression analysis revealed that lymph node metastases (OS, *P* = 0.047; DFS, *P* = 0.036) and TNM stage (OS, *P* = 0.034/0.006; DFS, *P* = 0.048/0.040) were prognostic factors for a shorter OS and DFS in BC patients.

To evaluate whether AIFM3 expression could serve as a predictive marker for breast cancer, we used ROC (receiver operating characteristic curve) analysis. The ROC curves displayed a discrimination of the expression levels of AIFM3 by OS. ROC yielded an AUC of 0.718 for AIFM3, with diagnostic value (*P* < 0.001, Additional file 3: Figure S2). Based on this outcome, AIFM3 had a predictive value for patient overall survival in breast cancer.

### AIFM3-related signaling pathways

**Fig. 3:**
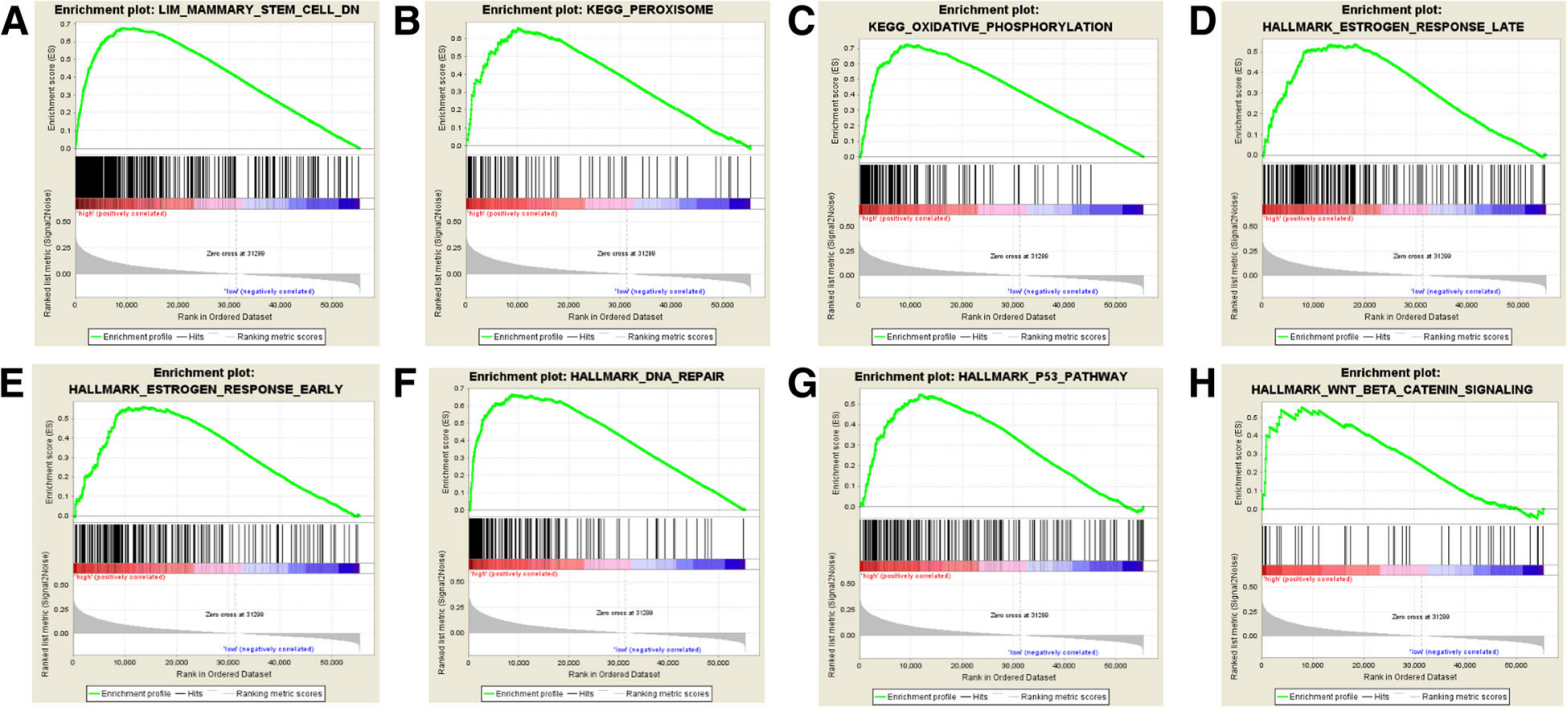
Enrichment plots from Gene Set Enrichment Analysis (GSEA). GSEA was used to indicate the mechanisms related to AIFM3 expression in BC. GSEA disclosed a significant enrichment of (**a**) Mammary stem cell-DN. **b** Peroxisome. **c** Oxidative phosphorylation. **d** Estrogen response late. **e** Estrogen response early. **f** DNA repair. **g** P53 pathway. **h** Wnt/β-catenin signaling

Significant enriched pathways were related to BC biological process according to NES (Fig. 3a-h and Table 3). TCGA-BRCA samples in high AIFM3 expression group was enriched in estrogen response (late and early), peroxisome, oxidative phosphorylation, DNA repair, P53 pathway, Wnt/β-Catenin pathway signaling, etc.

**Table 3:** Gene set enriched with *AIFM3* high expression

MsigDB collection	Gene set name	NES	NOM *p*-val	FDR *q*-val
c2.cgp.v6.2.symbols.gmt	LIM_MAMMARY_STEM_CELL_DN	2.226	0.000	0.008
c2.cp.kegg.v6.2.symbols.gmt	KEGG_PEROXISOME	2.120	0.000	0.004
	KEGG_OXIDATIVE_PHOSPHORYLATION	1.972	0.002	0.013
h.all.v6.0.symbols.gmt	HALLMARK_ESTROGEN_RESPONSE_LATE	2.040	0.000	0.030
	HALLMARK_ESTROGEN_RESPONSE_EARLY	2.000	0.002	0.022
	HALLMARK_DNA_REPAIR	1.917	0.004	0.015
	HALLMARK_P53_PATHWAY	1.862	0.002	0.020
	HALLMARK_WNT_BETA_CATENIN_SIGNALING	1.679	0.023	0.065

*NES* normalized enrichment score, *NOM* nominal, *FDR* false discovery rate

### Homology modeling of AIFM3

**Fig. 4:**
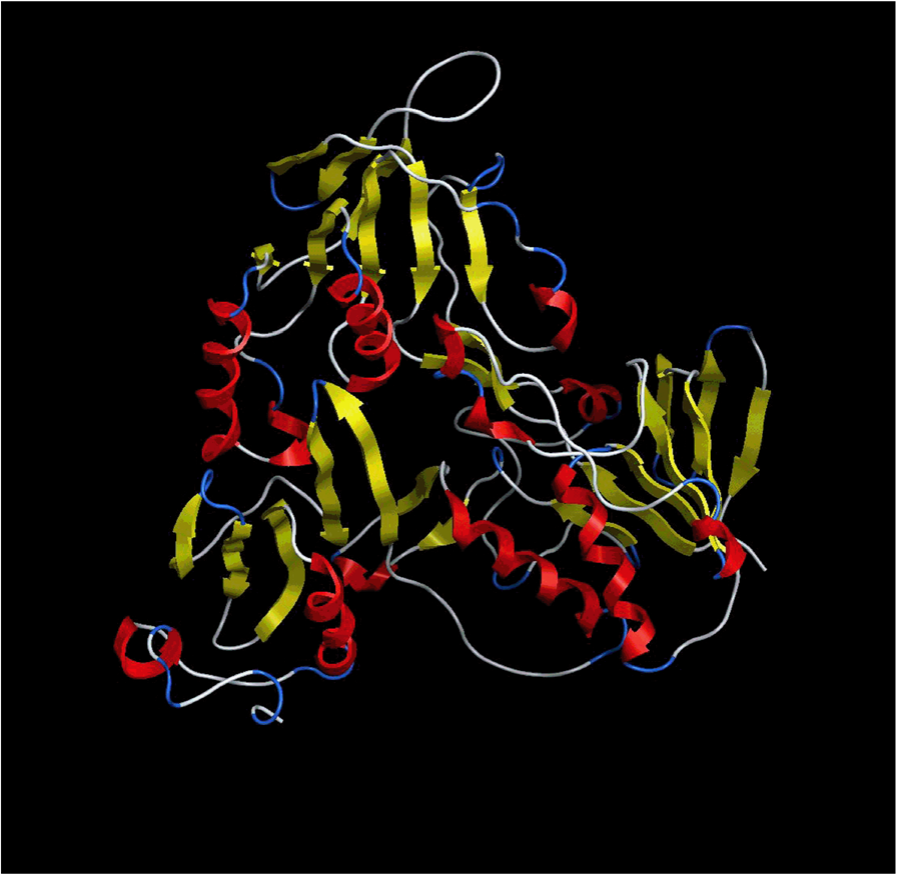
Homology modeling structure of AIFM3 by MOE. MOE software package 2018 contains user interface enhancements for protein modeling. Ribbon diagram of AIFM3 has four characteristic domains: turn (blue), helix (red), β-fold (yellow) and loop area (white)

We modelled the three-dimensional structure of human AIFM3 by certain X-ray crystal structure. The structure of toluene 2, 3-dioxygenase reductase (PDB ID: 3EF6) was selected as the template to build homology model of AIFM3, according to the highest sequence identity scores (33%, Additional file 4: Figure S3A). The sequences alignment of the newly-built human AIFM3 and 3EF6 was shown in Additional file 4: Figure S3B. Most residues of final models were in allowed regions of Ramachandran map (Fig. 4 and Additional file 4: Figure S3C).

### Protein AIFM3 - PTPN12 dock and expression correlation

**Fig. 5:**
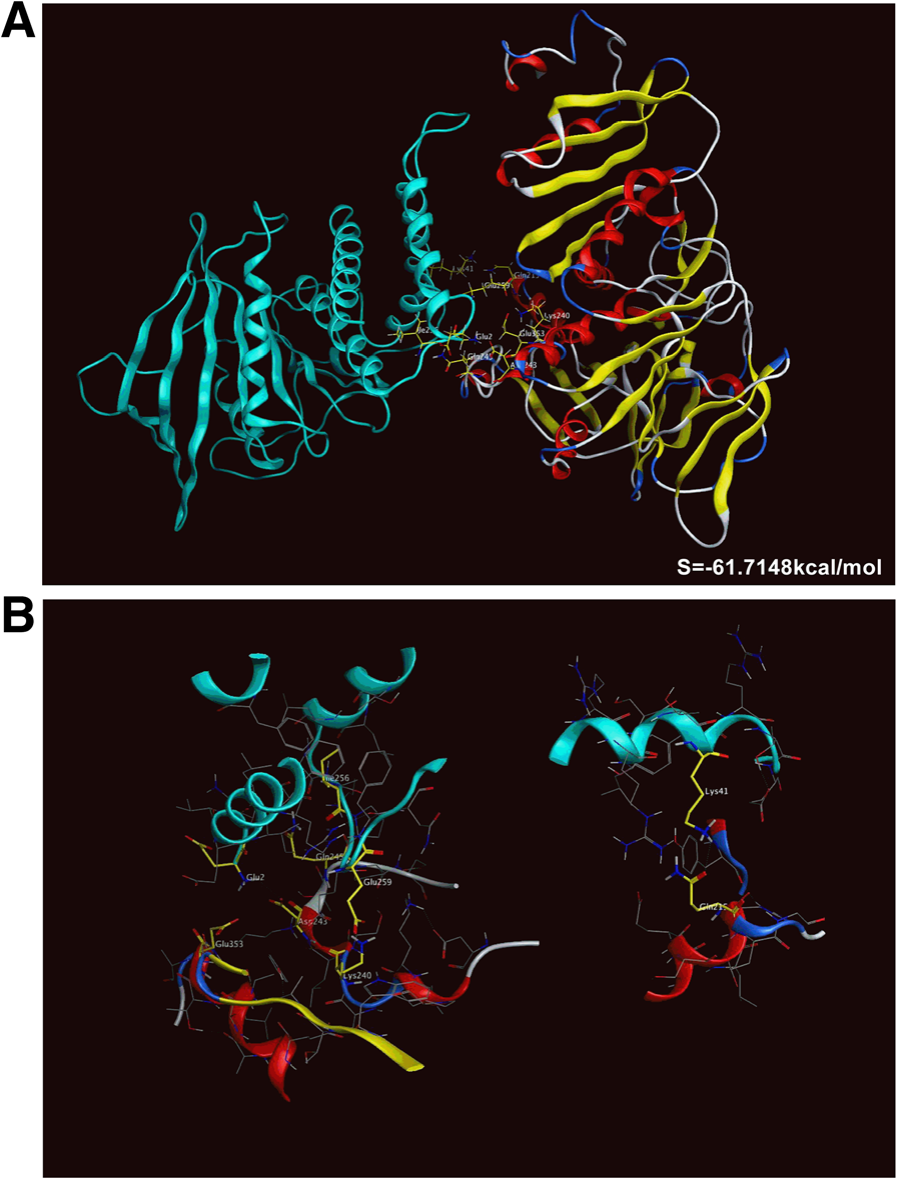
Protein-protein dock of AIFM3 and PTPN12 by MOE. MOE software package 2018 contains user interface enhancements for identifying the intramolecular interactions of proteins. **a** Ribbon representation of the three-dimensional (3D) interface between PTPN12 (blue) and AIFM3 (combination with red, blue white and yellow), with binding energy of − 61.7148 kcal/mol. **b** Binding sites of PTPN12 and AIFM3 dock and the residues marked by yellow

We searched the crystal structure of PTPN12 (PDB ID: 5HDE) in Protein Data Bank. MOE 2018 was used to dock protein AIFM3 and PTPN12.The lowest potential docking energy of AIFM3 and PTPN12 was − 61.71 kcal/mol (Fig. 5a). Residue E2 of PTPN12 was bound to residue D243 by hydrogen bond and induced force. Residue E2 of PTPN12, residue E259 of PTPN12 and residue K240 of AIFM3 had induced force. Besides, residue K42 of PTPN12 and residue Q240 of AIFM3, residue I259 of PTPN12 and residue E245 of AIFM3 had interaction by hydrogen bonds (Fig. 5b and Table 4). The interaction of residues of two proteins revealed that AIFM3 bond to PTPN12.

**Table 4:** AIFM3 and PTPN12 protein bonding contact

Type	ChainA	PosA	SetA	ChainB	PosB	SetB	Energy (kcal/mol)	Distant (A)
IH	2-5HDE.A	1	Glu2	1-AIFM3	49	Asp243	−11.21	3.033
I	2-5HDE.A	1	Glu2	1-AIFM3	159	Glu353	−3.74	3.117
H	2-5HDE.A	40	Lys41	1-AIFM3	21	Gln215	−2.3	2.965
H	2-5HDE.A	255	Ile256	1-AIFM3	51	Gln245	−0.7	3.29
I	2-5HDE.A	258	Glu259	1-AIFM3	46	Lys240	−5.639	2.84

*I* Inducing force, *H* Hydrogen bonding force, *IH* Inducing force and Hydrogen bonding force

IHC of PTPN12 was performed and reported by our research group. The graded staining intensity was shown in the previous article [15]. High and low expression of PTPN12 was shown in Additional file 5: Figure S4A-B. In 151 BC tissue specimens, both AIFM3 and PTPN were high-expressed in 63 cases (41.7%) and both were low-expressed in 39 cases (25. 8%). High AIFM3 and low PTPN12 expression were assessed in 32 cases (21.2%), while low AIFM3 and high PTPN12 expression were detected in 17 cases (11.2%). Spearman correlation analysis showed that AIFM3 was positively correlated with PTPN12. Spearman rs were 0.348 (*P* < 0.001).

## Discussion

Mitochondrial proteins played key roles in carcinogenesis of various cancer [16]. The expression levels of the mitochondrial proteins are found to be related to the progression of cancers, which warrants future investigation [17–19]. The studies of AIFM3 in cancer are limited to several cancers. AIFM3 was overexpressed in human CCA tissues. AIFM3 was a direct target of miR-210 which was related to proliferation of human hepatoma cells [20, 21]. So far, expression of AIFM3 has not been reported in BC. TCGA database analysis illustrated that the expression of AIFM3 was significantly higher in BC than in adjacent normal tissues. Our results were consistent with that found in TCGA, indicating that AIFM3 overexpression might facilitate malignant transformation and played an important role in the development and progression of BC. AIFM3 expression was associated with tumor size, lymph node metastasis, TNM stage and molecular typing. Lymph node metastasis and TNM stage were independent factors of AIFM3 expression. These results suggested that overexpression of AIFM3 predicted more proliferative and aggressive behavior of BC.

Until now, the relationship between AIFM3 expression and patient survival in BC has not been identified. Based on bioinformatics analysis of data mining and the sample data collected, this study found that higher AIFM3 expression at gene and protein level indicated a shorter OS and DFS over 5 years, by multiple statistical methods. The result indicated that AIFM3 might be involved in the postoperative recurrence or distant metastasis of BC. In the univariate analysis, AIFM3, tumor size, lymph node involvement, HER2-status and TNM stage are correlated with a worse prognosis. We included variables with statistical significance (*P* < 0.05) in the multivariate analysis, lymph node metastases and TNM stage were prognostic factors for a shorter OS and DFS in BC patients (*P* < 0.05). The *P* value of AIFM3 is 0.053, which may be due to the insufficient sample size. In ROC analysis of OS, the AUC reached 0.718, indicating a good predictive value for AIFM3 (*P* < 0.001). AIFM3 may be a candidate marker assisting survival prediction in clinical practice. Further studies in larger scale of patients and in-depth analysis are required to elucidate the prognostic value of AIFM3 in BC, especially the role of AIFM3 as a prognostic factor, in BC patients or in patients with various kinds of molecular typing.

The occurrence and development of malignant tumors is resulted by a variety of signal pathways together [22]. The present study identified the potentially related mechanisms that AIFM3 might influence BC development. From GSEA, high AIFM3 expression was enriched in several gene sets. AIFM3 might exert late and early response to estrogen and decrease stem-like properties of breast cancer cells and stemness of breast cancer stem cells. AIFM3 might be involved in tumor cell survival, proliferation, invasion and migration via P53 signal pathway and Wnt/β-catenin signal pathway [23, 24]. AIFM3 was relevant to oxidative phosphorylation, which indicated AIFM3 might participate in maintaining the energy metabolism of tumor cells. AIFM3 correlated to DNA repair and peroxisome, which indicated AIFM3 might participated in reactive oxygen species pathway to regulate cancer development [25].These results provides new insights for understanding the molecular mechanism of AIFM3 in regulating malignant tumor biology process. Since the molecular function of AIFM3 has not been fully explored, further studies are required to elucidate its role in carcinogenesis and metastasis.

We propose AIFM3 as a potential therapeutic target. It is theoretically possible for several reasons. Firstly, AIFM3 is more expressed in breast cancer tissue than in normal tissues. There is a significant association of AIFM3 expression with tumor size, lymph node metastasis, TNM staging and other clinical pathology factors, indicating that AIFM3 may be related to the occurrence and development of BC. Also, BC patients with high AIFM3 expression has poor prognosis. The result is a premise for AIFM3 to be a therapeutic target. Secondly, from GSEA, we propose AIFM3 may decrease stem-like properties of breast cancer cells and stemness of breast cancer stem cells (BCSCs). AIFM3 is also related to Wnt/β-catenin signal pathway, a recognized pathway in regulating the self-renewal of BCSCs. BCSCs, characterized by self-renewal and pluripotency, are regarded as the source of drug resistance and recurrence in BC. The use of stem cells and targeting the signaling pathway in therapy has shown attractive prospects. AIFM3 may have the potential to suppress tumor via targeting BCSCs.

AIFM3 plays a major role in caspase-dependent apoptosis [26]. AIFM3 containes an additional 2Fe-2S Rieske domain, which may be important for apoptosis induction. AIFM3 needs additional partners to fulfill its apoptogenic function. So it makes sense to study whether AIFM3 can interact with other proteins during apoptosis initiation and execution. PTPN12 facilitates cellular detachment by acting on an unidentified substrate and activating caspase-3 in cell death signal [27]. Caspase-3-cleaved form of PTPN12 controls EphA3 phosphorylation and ephrin-induced cytoskeletal remodeling [28]. PTPN12 has an N′-terminal phosphatase domain, as well as a C′-terminal region which contains multiple poly-proline rich sequences, contributing to substrate specificity through the protein–protein interaction [29]. Here, we used a novel software MOE 2018 to model the crystal structure of AIFM3 and docked protein PTPN12 onto AIFM3. The present study predicted binding residues of two proteins and revealed that crystal structure of AIFM3 bonded to PTPN12. We add to growing evidence that AIFM3 may interact with PTPN12. However, the mechanism of AIFM3 induced-apoptosis needs more study supported by experiments. Further study will be performed to determine interactions of AIFM3 and PTPN12.

## Conclusion

AIFM3 was significantly more expressed in breast cancer tissues than in normal tissues. There was a significant association of AIFM3 expression with tumor size, lymph node metastasis, molecular typing and TNM staging. Lymph node metastasis and TNM stage were independent factors of AIFM3 expression. High AIFM3 expression was related to a shorter OS and DFS. AIFM3 might be closely related to occurrence and development of breast cancer.

## Additional files

Additional file 1: **Figure S1.** Dot plot of AIFM3 levels in breast cancer. AIFM3 was classified into “high” and “low” AIFM3-expression groups in TCGA (*P* < 0.0001). (TIF 1211 kb)

Additional file 2: **Table S1.** Univariable and multivariable analysis of overall survival in breast cancer patients. **Table S2.** Univariable and multivariable analysis of disease-free survival in breast cancer patients. (DOCX 22 kb)

Additional file 3: **Figure S2.** The discrimination of AIFM3 levels by OS in ROC analysis. ROC yielded an AUC of 0.718 for AIFM3, with diagnostic value (*P* < 0.001). (TIF 3914 kb)

Additional file 4: **Figure S3.** Process of homology modeling of AIFM3. (A) The identity of AIFM3 sequence and sequences of proteins in PDB analyzed by NCBI BLAST. (B) Alignment of AIFM3 sequence and 3EF6. The color of residue column based on degree of conservation between the sequences of AIFM3 and 3EF6. The degree colored from red (not conserved) to blue (fully conserved). (C) Ramachandran map of residues of AIFM3. (TIF 2359 kb)

Additional file 5: **Figure S4.** Expression level of PTPN12 in IHC. (A) High expression of PTPN12 (B) Low expression of PTPN12 (TIF 4791 kb)

## Abbreviations

AIFM3: Apoptosis-Inducing Factor Mitochondrion-associated 3
BC: Breast cancer
CCA: Cholangiocarcinoma
DFS: Disease-free survival
GSEA: Gene set enrichment analysis
IHC: Immunohistochemistry
MOE: Molecular Operating Environment software
OS: Overall survival
PTPN12: Protein Tyrosine Phosphatase Nonreceptor-type12

## References

[1] Siegel RL, Miller KD, Jemal A (2018). Cancer statistics, 2018. CA Cancer J Clin.

[2] Fan L, Strasser-Weippl K, Li JJ, St Louis J, Finkelstein DM, Yu KD (2014). Breast cancer in China. Lancet Oncol.

[3] Li Y, Zhang H, Zhao Y, Wang C, Cheng Z, Tang L (2019). A mandatory role of nuclear PAK4-LIFR axis in breast-to-bone metastasis of ERalpha-positive breast cancer cells. Oncogene..

[4] Chavez-MacGregor M, Mittendorf EA, Clarke CA, Lichtensztajn DY, Hunt KK, Giordano SH (2017). Incorporating tumor characteristics to the American joint committee on Cancer breast Cancer staging system. Oncologist..

[5] Xie Q, Lin T, Zhang Y, Zheng J, Bonanno JA (2005). Molecular cloning and characterization of a human AIF-like gene with ability to induce apoptosis. J Biol Chem.

[6] Chua-On D, Proungvitaya T, Techasen A, Limpaiboon T, Roytrakul S, Wongkham S (2016). High expression of apoptosis-inducing factor, mitochondrion-associated 3 (AIFM3) in human cholangiocarcinoma. Tumour Biol.

[7] Yang W, Sun T, Cao J, Liu F, Tian Y, Zhu W (2012). Downregulation of miR-210 expression inhibits proliferation, induces apoptosis and enhances radiosensitivity in hypoxic human hepatoma cells in vitro. Exp Cell Res.

[8] Sun T, Aceto N, Meerbrey KL, Kessler JD, Zhou C, Migliaccio I (2011). Activation of multiple proto-oncogenic tyrosine kinases in breast cancer via loss of the PTPN12 phosphatase. Cell..

[9] Rupinder SK, Gurpreet AK, Manjeet S (2007). Cell suicide and caspases. Vasc Pharmacol.

[10] Halle M, Tremblay ML, Meng TC (2007). Protein tyrosine phosphatases: emerging regulators of apoptosis. Cell Cycle.

[11] Devlin C, Greco S, Martelli F, Ivan M (2011). miR-210: more than a silent player in hypoxia. IUBMB Life.

[12] Chen B, Tang H, Chen X, Zhang G, Wang Y, Xie X (2018). Transcriptomic analyses identify key differentially expressed genes and clinical outcomes between triple-negative and non-triple-negative breast cancer. Cancer Manag Res.

[13] Subramanian A, Tamayo P, Mootha VK, Mukherjee S, Ebert BL, Gillette MA (2005). Gene set enrichment analysis: a knowledge-based approach for interpreting genome-wide expression profiles. Proc Natl Acad Sci U S A.

[14] Gentile F, Barakat KH, Tuszynski JA (2018). Computational characterization of small molecules binding to the human XPF active site and virtual screening to identify potential new DNA repair inhibitors targeting the ERCC1-XPF endonuclease. Int J Mol Sci.

[15] Wang YY, Liu H, Mao XY, Jin F, Ma B, Jiang JY (2016). Identifying the role of PTPN12 expression in predicting the efficacy of capecitabine to neoadjuvant chemotherapy in breast cancer treatment. Eur Rev Med Pharmacol Sci.

[16] Srinivasan S, Guha M, Kashina A, Avadhani NG (2017). Mitochondrial dysfunction and mitochondrial dynamics-the cancer connection. Biochim Biophys Acta Bioenerg.

[17] McGee AM, Douglas DL, Liang Y, Hyder SM, Baines CP (2011). The mitochondrial protein C1qbp promotes cell proliferation, migration and resistance to cell death. Cell Cycle.

[18] Jain M, Nilsson R, Sharma S, Madhusudhan N, Kitami T, Souza AL (2012). Metabolite profiling identifies a key role for glycine in rapid cancer cell proliferation. Science..

[19] Wu G, Zhou W, Pan X, Sun Y, Xu H, Shi P (2018). miR-100 reverses cisplatin resistance in breast Cancer by suppressing HAX-1. Cell Physiol Biochem.

[20] Dang K, Myers KA (2015). The role of hypoxia-induced miR-210 in cancer progression. Int J Mol Sci.

[21] Wang L, Yue Y, Wang X, Jin H (2015). Function and clinical potential of microRNAs in hepatocellular carcinoma. Oncol Lett.

[22] Hanahan D, Weinberg RA (2011). Hallmarks of cancer: the next generation. Cell..

[23] Shen X, Zhang Y, Wu X, Guo Y, Shi W, Qi J (2017). Upregulated lncRNA-PCAT1 is closely related to clinical diagnosis of multiple myeloma as a predictive biomarker in serum. Cancer Biomark.

[24] Thai P, Statt S, Chen CH, Liang E, Campbell C, Wu R (2013). Characterization of a novel long noncoding RNA, SCAL1, induced by cigarette smoke and elevated in lung cancer cell lines. Am J Respir Cell Mol Biol.

[25] Ali R, Rakha EA, Madhusudan S, Bryant HE (2017). DNA damage repair in breast cancer and its therapeutic implications. Pathology..

[26] Yang W, Wei J, Sun T, Liu F (2013). Effects of knockdown of miR-210 in combination with ionizing radiation on human hepatoma xenograft in nude mice. Radiat Oncol.

[27] Halle M, Liu YC, Hardy S, Theberge JF, Blanchetot C, Bourdeau A (2007). Caspase-3 regulates catalytic activity and scaffolding functions of the protein tyrosine phosphatase PEST, a novel modulator of the apoptotic response. Mol Cell Biol.

[28] Mansour M, Nievergall E, Gegenbauer K, Llerena C, Atapattu L, Halle M (2016). PTP-PEST controls EphA3 activation and ephrin-induced cytoskeletal remodelling. J Cell Sci.

[29] Arimura Y, Shimizu K, Koyanagi M, Yagi J (2014). Effects of protein tyrosine phosphatase-PEST are reversed by Akt in T cells. Cell Signal.

