# Supplementary figures and images for: Clinical implications of a novel prognostic factor AIFM3 in breast cancer patients
Source: BMC Cancer. 2019 May 14;19:451. doi: 10.1186/s12885-019-5659-4 (PMC6518782; doi:10.1186/s12885-019-5659-4)

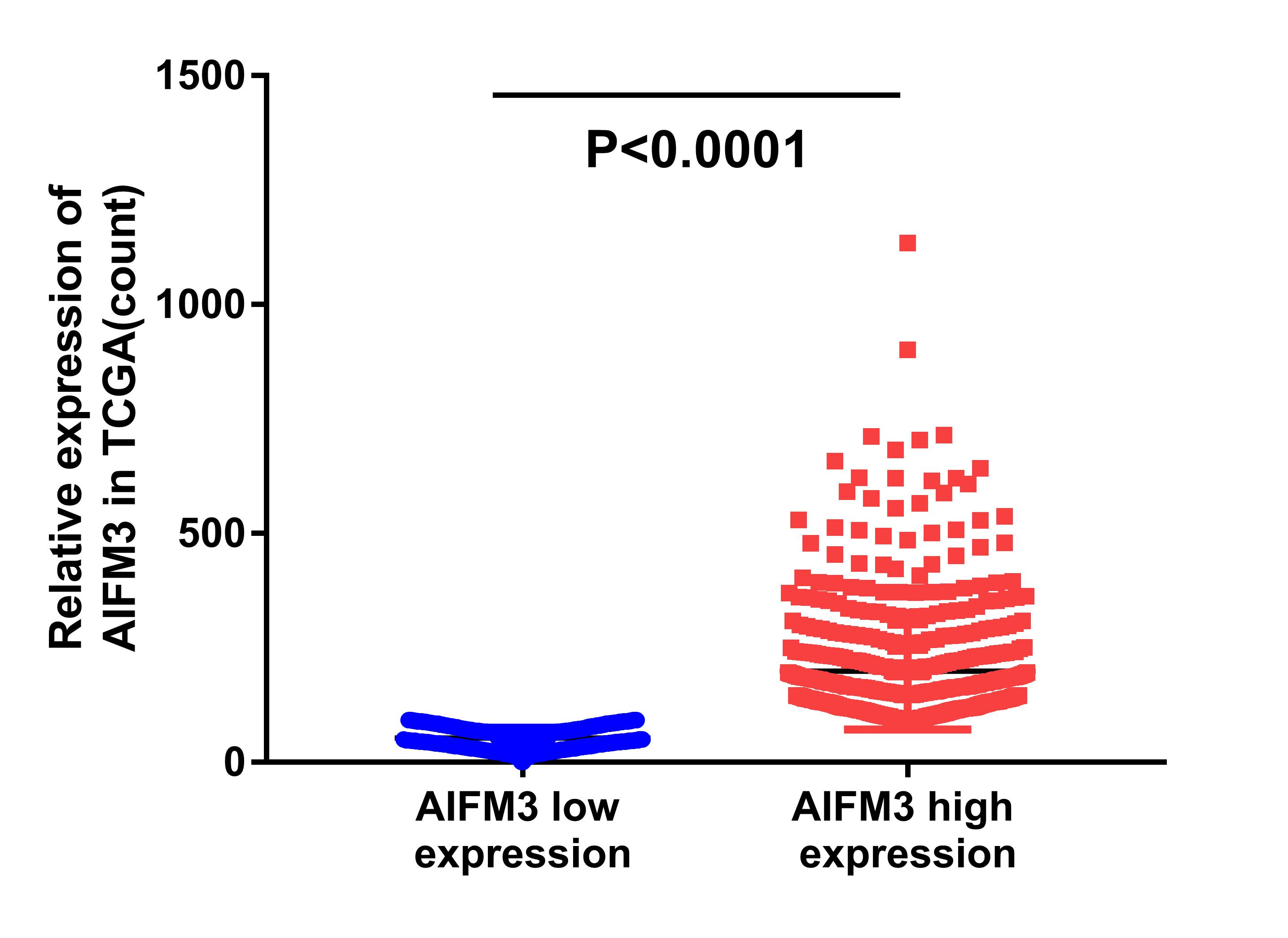

Supplement: Supplementary file 1 — Figure S1. Dot plot of AIFM3 levels in breast cancer. AIFM3 was classified into “high” and “low” AIFM3-expression groups in TCGA (P < 0.0001). (TIF 1211 kb) [file 12885_2019_5659_MOESM1_ESM.tif]

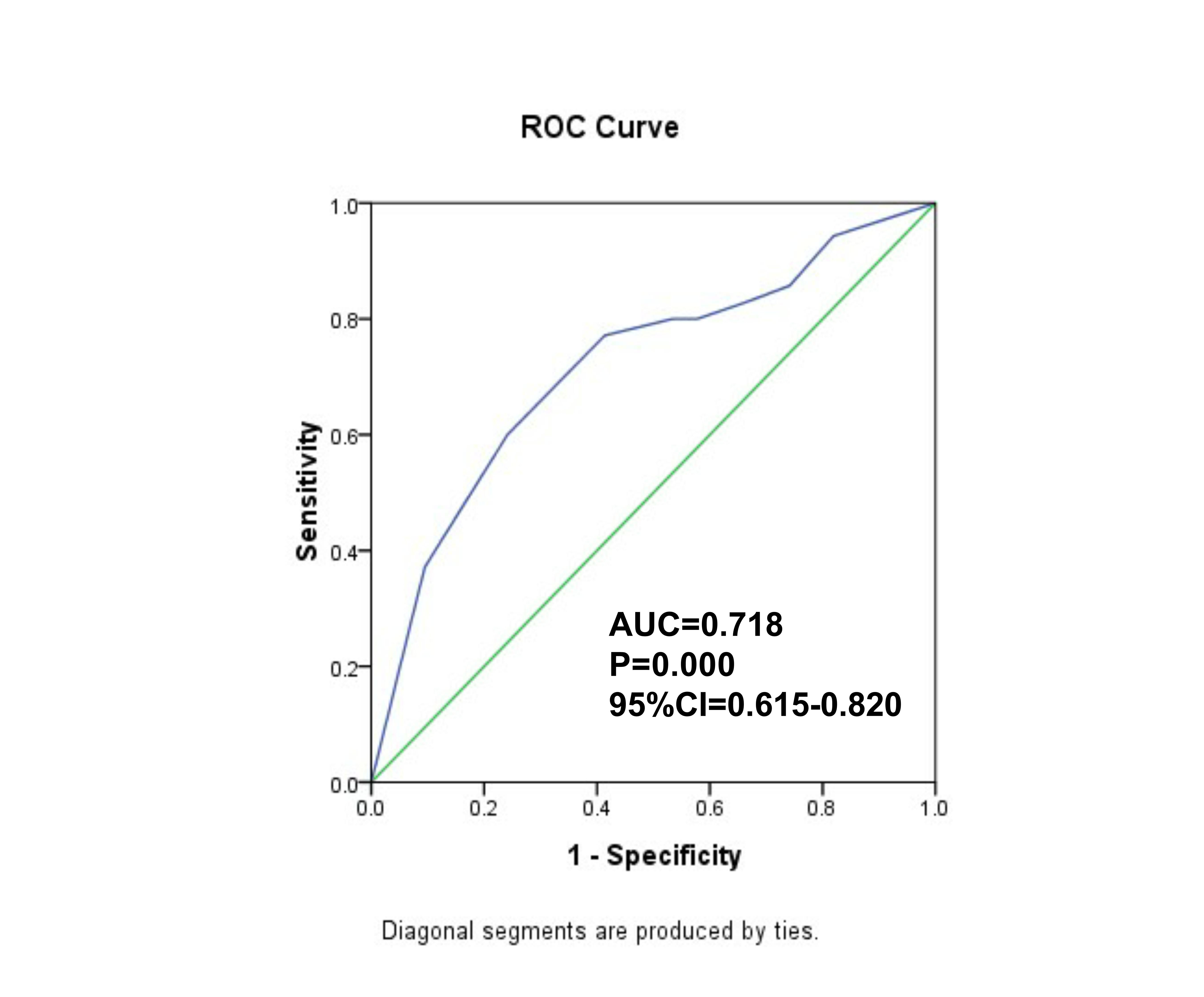

Supplement: Supplementary file 3 — Figure S2. The discrimination of AIFM3 levels by OS in ROC analysis. ROC yielded an AUC of 0.718 for AIFM3, with diagnostic value (P < 0.001). (TIF 3914 kb) [file 12885_2019_5659_MOESM3_ESM.tif]
