# Supplementary material for: Clinical implications of a novel prognostic factor AIFM3 in breast cancer patients
Source: BMC Cancer. 2019 May 14;19:451. doi: 10.1186/s12885-019-5659-4 (PMC6518782; doi:10.1186/s12885-019-5659-4)
Supplement: Supplementary file 2 — Table S1. Univariable and multivariable analysis of overall survival in breast cancer patients. Table S2. Univariable and multivariable analysis of disease-free survival in breast cancer patients. (DOCX 22 kb) [file 12885_2019_5659_MOESM2_ESM.docx]

| **Table S1. Univariable and multivariable analysis of overall survival** | | | | | | | |
| --- | --- | --- | --- | --- | --- | --- | --- |
| **Factors** | **Univariate Analysis** | | | **Multivariate Analysis** | | | |
|  | **P** | **HR** | **95 CI** | **P** | **Adjusted HR** | **95 CI** |  |
| **Age(years)** |  |  |  |  |  |  |  |
| **≤ 40** | 0.934 | 1.043 | 0.388-2.800 |  |  |  |  |
| **41-50** | 0.364 | 0.659 | 0.268-1.622 |  |  |  |  |
| **51-60** | 0.643 | 0.804 | 0.319-2.025 |  |  |  |  |
| **≥61** | Reference |  |  |  |  |  |  |
| **Tumor Size** |  |  |  |  |  |  |  |
| **≥ 3 cm** | 0.003 | 3.464 | 1.512-7.933 | 0.566 | 0.679 | 0.181-2.545 |  |
| **< 3 cm** | Reference |  |  | Reference |  |  |  |
| **LN Metastases** |  |  |  |  |  |  |  |
| **negative** | Reference |  |  | Reference |  |  |  |
| **positive** | <0.001 | 5.688 | 2.582-12.533 | 0.047 | 4.262 | 1.018-17.835 |  |
| **ER** |  |  |  |  |  |  |  |
| **negative** | Reference |  |  | Reference |  |  |  |
| **positive** | 0.027 | 2.432 | 1.105-5.355 | 0.405 | 0.601 | 0.181-1.991 |  |
| **PR** |  |  |  |  |  |  |  |
| **negative** | Reference |  |  |  |  |  |  |
| **positive** | 0.818 | 1.084 | 0.546-2.151 |  |  |  |  |
| **Her2** |  |  |  |  |  |  |  |
| **negative** | Reference |  |  | Reference |  |  |  |
| **positive** | 0.006 | 2.518 | 1.297-4.889 | 0.272 | 1.579 | 0.699-3.567 |  |
| **AIFM3** |  |  |  |  |  |  |  |
| **High Expression** | 0.022 | 2.631 | 1.149-6.026 | 0.053 | 2.477 | 0.990-6.198 |  |
| **Low Expression** | Reference |  |  | Reference |  |  |  |
| **Histological grade** |  |  |  |  |  |  |  |
| **2** | Reference |  |  |  |  |  |  |
| **3** | 0.991 | 1.007 | 0.306-3.312 |  |  |  |  |
| **unrated** | 0.539 | 1.389 | 0.487-3.960 |  |  |  |  |
| **Molecular typing** |  |  |  |  |  |  |  |
| **Luminal A** | 0.743 | 0.818 | 0.246-2.717 |  |  |  |  |
| **Luminal B** | 0.035 | 3.181 | 1.086-9.312 |  |  |  |  |
| **Her-2** | 0.981 | 0.982 | 0.220-4.387 |  |  |  |  |
| **TNBC** | Reference |  |  |  |  |  |  |
| **TNM staging** |  |  |  |  |  |  |  |
| **I** | Reference |  |  | Reference |  |  |  |
| **II** | 0.027 | 9.680 | 1.288-72.746 | 0.034 | 10.170 | 1.195-85.560 |  |
| **III** | 0.003 | 22.375 | 2.976-168.230 | 0.006 | 20.552 | 2.424-174.266 |  |

| **Table S2. Univariable and multivariable analysis of disease-free survival** | | | | | | | |
| --- | --- | --- | --- | --- | --- | --- | --- |
| **Factors** | **Univariate Analysis** | | | **Multivariate Analysis** | | | |
|  | **P** | **HR** | **95 CI** | **P** | **Adjusted HR** | **95 CI** |  |
| **Age(years)** |  |  |  |  |  |  |  |
| **≤ 40** | 0.815 | 1.111 | 0.460-2.682 |  |  |  |  |
| **41-50** | 0.794 | 0.903 | 0.419-1.945 |  |  |  |  |
| **51-60** | 0.996 | 0.998 | 0.447-2.227 |  |  |  |  |
| **≥61** | Reference |  |  |  |  |  |  |
| **Tumor size** |  |  |  |  |  |  |  |
| **≥ 3 cm** | <0.001 | 3.494 | 1.743-7.004 | 0.594 | 1.346 | 0.452-4.010 |  |
| **< 3 cm** | Reference |  |  | Reference |  |  |  |
| **LN Metastases** |  |  |  |  |  |  |  |
| **negative** | Reference |  |  | Reference |  |  |  |
| **positive** | <0.001 | 3.847 | 2.093-7.072 | 0.036 | 2.627 | 1.063-6.493 |  |
| **ER** |  |  |  |  |  |  |  |
| **negative** | Reference |  |  | Reference |  |  |  |
| **positive** | 0.007 | 2.534 | 1.295-4.959 | 0.530 | 0.727 | 0.269-1.966 |  |
| **PR** |  |  |  |  |  |  |  |
| **negative** | Reference |  |  |  |  |  |  |
| **positive** | 0.740 | 1.103 | 0.617-1.972 |  |  |  |  |
| **Her2** |  |  |  |  |  |  |  |
| **negative** | Reference |  |  |  |  |  |  |
| **positive** | 0.131 | 1.550 | 0.877-2.741 |  |  |  |  |
| **AIFM3** |  |  |  |  |  |  |  |
| **High Expression** | 0.034 | 2.019 | 1.053-3.874 | 0.106 | 1.782 | 0.884-3.595 |  |
| **Low Expression** | Reference |  |  | Reference |  |  |  |
| **Histological grade** |  |  |  |  |  |  |  |
| **2** | Reference |  |  |  |  |  |  |
| **3** | 0.566 | 0.709 | 0.219-2.294 |  |  |  |  |
| **unrated** | 0.105 | 1.948 | 0.871-4.358 |  |  |  |  |
| **Molecular typing** |  |  |  |  |  |  |  |
| **Luminal A** | 0.435 | 1.483 | 0.551-3.995 |  |  |  |  |
| **Luminal B** | 0.050 | 2.642 | 1.000-6.978 |  |  |  |  |
| **Her-2** | 0.987 | 0.989 | 0.266-3.684 |  |  |  |  |
| **TNBC** | Reference |  |  |  |  |  |  |
| **TNM staging** |  |  |  |  |  |  |  |
| **I** | Reference |  |  | Reference |  |  |  |
| **II** | 0.022 | 3.069 | 1.178-7.995 | 0.048 | 2.870 | 1.009-8.168 |  |
| **III** | 0.002 | 4.804 | 1.782-12.948 | 0.040 | 3.242 | 1.053-9.982 |  |
